# Implementation of the Good School Toolkit in Uganda: a quantitative process evaluation of a successful violence prevention program

**DOI:** 10.1186/s12889-018-5462-1

**Published:** 2018-05-09

**Authors:** Louise Knight, Elizabeth Allen, Angel Mirembe, Janet Nakuti, Sophie Namy, Jennifer C. Child, Joanna Sturgess, Nambusi Kyegombe, Eddy J. Walakira, Diana Elbourne, Dipak Naker, Karen M. Devries

**Affiliations:** 1 London School of Hygiene and Tropical Medicine, Keppel Street, London, WC1E 7HT UK; 2 Raising Voices, Kampala, Uganda; 3 Makerere University, Kampala, Uganda

**Keywords:** Process evaluation, Violence, Schools, Children, Corporal punishment, Uganda

## Abstract

**Background:**

The Good School Toolkit, a complex behavioural intervention designed by Raising Voices a Ugandan NGO, reduced past week physical violence from school staff to primary students by an average of 42% in a recent randomised controlled trial. This process evaluation quantitatively examines what was implemented across the twenty-one intervention schools, variations in school prevalence of violence after the intervention, factors that influence exposure to the intervention and factors associated with students’ experience of physical violence from staff at study endline.

**Methods:**

Implementation measures were captured prospectively in the twenty-one intervention schools over four school terms from 2012 to 2014 and Toolkit exposure captured in the student (*n* = 1921) and staff (*n* = 286) endline cross-sectional surveys in 2014. Implementation measures and the prevalence of violence are summarised across schools and are assessed for correlation using Spearman’s Rank Correlation Coefficient. Regression models are used to explore individual factors associated with Toolkit exposure and with physical violence at endline.

**Results:**

School prevalence of past week physical violence from staff against students ranged from 7% to 65% across schools at endline. Schools with higher mean levels of teacher Toolkit exposure had larger decreases in violence during the study. Students in schools categorised as implementing a ‘low’ number of program school-led activities reported less exposure to the Toolkit. Higher student Toolkit exposure was associated with decreased odds of experiencing physical violence from staff (OR: 0.76, 95%CI: 0.67-0.86, *p*-value< 0.001). Girls, students reporting poorer mental health and students in a lower grade were less exposed to the toolkit. After the intervention, and when adjusting for individual Toolkit exposure, some students remained at increased risk of experiencing violence from staff, including, girls, students reporting poorer mental health, students who experienced other violence and those reporting difficulty with self-care.

**Conclusions:**

Our results suggest that increasing students and teachers exposure to the Good School Toolkit within schools has the potential to bring about further reductions in violence. Effectiveness of the Toolkit may be increased by further targeting and supporting teachers’ engagement with girls and students with mental health difficulties.

**Trial registration:**

The trial is registered at clinicaltrials.gov, NCT01678846, August 24th 2012.

**Electronic supplementary material:**

The online version of this article (10.1186/s12889-018-5462-1) contains supplementary material, which is available to authorized users.

## Background

In Uganda, physical punishment in schools has been banned since 1997, and became illegal in May 2016. Despite this, physical punishment persists as normal practice in primary schools. In one study conducted in 2012, over half of school children reported experiencing physical violence from staff in the last week and 8% sought treatment for injury from a healthcare provider [1]. This high level of violence in schools is not unique to Uganda. Recent national prevalence studies have shown that 40% of 13-17 year olds report being punched, kicked or whipped by a teacher in the last week in Kenya and in Tanzania 50% reported experiencing physical violence from a teacher when they were under 18 year of age [2, 3].

The Good School Toolkit developed by Raising Voices, a Uganda-based Non Government Organisation (NGO), is one of the very few rigorously evaluated interventions designed to reduce physical violence from school staff to students. The Toolkit is a violence prevention behavioural intervention that aims to change school operational culture. We recently conducted a trial to assess effectiveness as part of the Good Schools Study. The trial results showed a 42% reduction in relative risk of students experiencing physical violence in the last week from staff (corresponding to an odds ratio: 0.40, 95% CI: 0.26 to 0.64, *p* < 0.001) [4]. However, even with this highly effective intervention, 31% of students in the intervention schools had experienced physical violence from staff in the last week after intervention delivery. This may be due to variation in delivery of the intervention by Raising Voices, school-led Toolkit implementation by school staff, adoption of the intervention by schools, or due to the characteristics of schools or composition of students within schools.

Exploring reasons for variation in intervention impact in complex interventions such as the Good School Toolkit is important to inform intervention development, adaptations, program monitoring, cost-effective implementation and scale-up [5]. Qualitative and quantitative methods for evaluation bring complementary insights into what and how interventions are delivered and received. Quantitative evaluation can not only describe what was implemented but also explore dose response and how delivery and reach may vary across contexts or participant characteristics [6]. Quantitative evaluation can therefore add valuable insight into how implementation is associated with effectiveness, and can highlight inequalities to inform intervention future development. However, quantitative process evaluations measuring implementation are relatively rare, with a limited number of health related behaviour change interventions reporting on how implementation is related to outcomes [7–10].

Here we present a quantitative process evaluation of the Good School Toolkit intervention in Uganda that focuses on the Toolkit implementation and explaining variations in effectiveness across schools. In associated papers we present the study protocol [11], main study results [4], qualitative findings on pathways of change [12] and an economic evaluation of the Good School Toolkit [13].

### Objectives

**Fig. 1:**
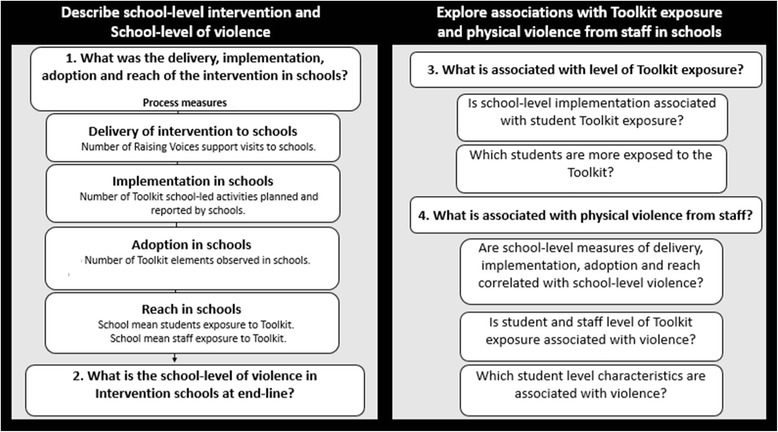
Summarises process evaluation objectives and specific questions addressed in this paper Legend for Fig. 1: Figure 1 summarises the four process evaluation objectives and the specific questions addressed in this paper. The left hand panel describes the school-level intervention and outcome, and lists the process measures explored in this analysis. The right hand panel describes exploratory analysis of factors associated with Toolkit exposure and the violence outcome

The specific process evaluation objectives are to: 1) describe measures of Toolkit delivery, implementation, adoption and reach in schools, 2) describe the prevalence of physical violence from staff across the intervention schools at baseline and at endline, 3) explore factors associated with student’s exposure to the Toolkit, and 4) explore factors (student and school-level) associated with physical violence from staff at endline. Figure 1 summarises the process evaluation objectives, Toolkit implementation process measures and sub-questions addressed in this paper.

## Methods

### Overview of main trial

The Good Schools Study consists of a cluster randomised controlled trial, a qualitative evaluation, an economic evaluation, and the process evaluation presented here. A cross-sectional baseline survey was conducted in June 2012, and endline survey during June 2014 in 42 primary schools in Luwero District, Uganda. Luwero is a large district with urban trading centres and rural sub-districts. Using school enrolment lists, 151 eligible primary schools were identified and grouped into strata according to the sex ratio of their students. Forty-two primary schools were randomly selected proportional to the stratum size, and all agreed to participate. Stratified randomisation was carried out after the baseline survey, with 21 schools receiving the Good School Toolkit and 21 forming a waitlisted control group. All 21 intervention schools completed the Toolkit intervention, which took place over 18 months (corresponding to four school terms) between September 2012 and May 2014. At endline, 92% of the sampled students were interviewed and 91% of all staff interviewed. The study was approved by the London School of Hygiene and Tropical Medicine Ethics Committee (6183) and the Uganda National Council for Science and Technology (SS2520). Our protocol and main trial results, that include details on ethics and consent procedures for all participants in the Good School Study, as well as child protection referral procedures, are published elsewhere [4, 11, 14].

### Intervention

The Good School Toolkit is publicly available at www.raisingvoices.org. It consists of a six-step process that involves implementation of a series of about 60 activities described in manuals and supporting leaflets and posters. Activities are coordinated by two lead teacher ‘protagonists’ and two student representatives in each school; some activities involve outreach to parents and the surrounding community. Schools receive one-on-one support visits and phone calls from Raising Voices staff. The intervention uses the Transtheoretical behaviour change model [15] and involves the application of behaviour change techniques shown to be effective in other fields and in other violence prevention interventions, including setting a goal, making an action plan, and providing social support [16]. A description of the Good School Toolkit intervention and a summary of the Toolkit six-step process are presented in Additional file 1.

### Process evaluation design

Drawing broadly on the Grant et al. 2013 process evaluation framework [17] we describe the overall implementation of the Toolkit in terms of four implementation components: Raising Voices delivery of the intervention to schools, school-led implementation of Toolkit activities, adoption of Toolkit elements in schools and the reach of the intervention to students and teachers at school. A pre-specified set of process data were prospectively captured in the 21 intervention schools during the 18-month implementation of the Toolkit and at the study end-line survey in 2014. All the measures were developed and pilot tested in the field before use and are described with data quality summarised in Additional file 2.

**Delivery of intervention to schools by Raising Voices** was measured using data routinely collected by Raising Voices Programme Officers as part of the intervention. Each of four officers directly supported five or six schools, and a Program Manager provided oversight and audit visits to the schools. All interactions with the schools—including technical support visits, group trainings and telephone calls—were systematically documented by each program officer termly. For analysis, the ‘delivery’ variable used was the number of Raising Voices technical support visits per school.

**School-led implementation of Toolkit activities** was measured using termly ‘action plans’ routinely completed by schools as part of the intervention, and standardised activity monitoring forms distributed to intervention schools collected only for the Good Schools Study. Schools were asked to complete action plans detailing all Toolkit activities they would conduct at the start of each term, and then to record the details of each activity they actually completed on a monitoring form (one monitoring form per activity). The number of planned and completed school-led activities reported were measured throughout implementation and used as the implementation process measures.

**Adoption of Toolkit elements by schools** was tracked by an independent ‘Study Process Monitor’ who was hired specifically to collect data on implementation of the intervention for the Good Schools Study. Once every term, she asked the Head Teacher or teacher protagonist a standard set of questions about Toolkit structures present in each school, a sub-set of which were verified by direct observation (e.g., the presence of a Good School mural). The number of Toolkit elements present in school were measured using data from the last term of implementation and used as the adoption process measure. This multi-item measure was constructed based on fourteen observations (count 0-14, Cronbach alpha 0.72 that are listed in Additional file 2).

**Fig. 2:**
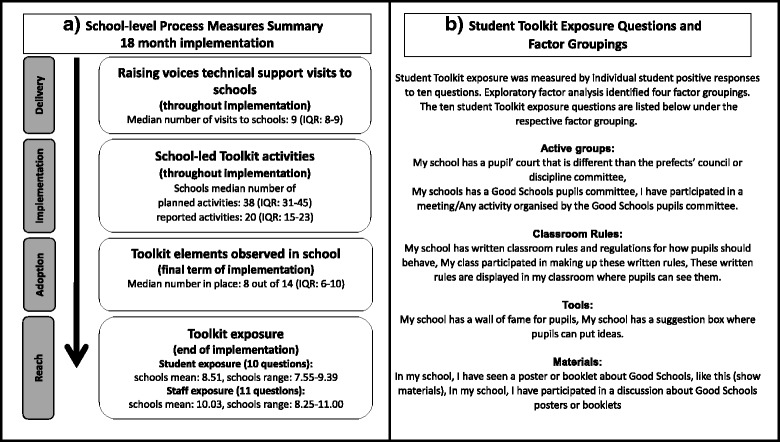
Figure 2a: Delivery, implementation, adoption and reach in the intervention schools. Figure 2b: Student Toolkit exposure questions and factor groupings Notation: IQR: Interquartile range, range: full range

**Toolkit reach to students and teachers in schools** was measured as school level aggregate of individual Toolkit exposure, using data from the 2014 endline surveys, conducted as part of the Good Schools Study. The surveys included a set of 10 student questions and 11 school staff questions on exposure to the Toolkit that captured awareness and participation in various Toolkit activities and processes. Student exposure questions are listed in Fig. 2b and include, “My schools has a Good Schools pupils committee.” Staff questions include “My school has a suggestion box where pupils can put ideas” and “My school has a Good Schools staff committee”. A full list of staff questions is available in Additional file 2. Binary yes or no responses to exposure questions were constructed as a count 0-10 and 0-11 for students and staff respectively, with a higher score representing more exposure. The staff exposure score had high internal reliability as measured by Cronbach’s alpha 0.72. Staff surveyed were 87% teaching staff and 13% administrators, cooks and other staff. The student exposure score had a low Cronbach’s alpha of 0.56 and therefore exploratory factor analysis was performed on student exposure responses. We used tetrachoric correction to account for binary variables, we retained factors with eigenvalues above 1 and Promax rotation was performed [18, 19]. The resulting predicted estimations were generated for each of factor groupings and summed for the total student Toolkit exposure score. The exploratory factor analyses identified four factor groupings that represented exposure to Good School Toolkit: 1) active groups, 2) classroom rules, 3) tools and 4) materials (items described in Fig. 2b).

**Other factors** were measured using items from the baseline and endline surveys, described elsewhere [1, 4]. Violence exposure (in students) and use (in staff) was measured using an adapted version of the International Society for the Prevention of Child Abuse and Neglect Child Abuse Screening Tool-Child Institutional (ICAST) [20]. Mental health difficulties were measured using the Strengths and Difficulties Questionnaire (SDQ) [21]. Both these measures have been widely used and have established data on aspects of validity and reliability in different contexts [20–25]. All questions were adapted and piloted in the local population prior to use in the survey.

### Data management

The Study Process Monitor was responsible for all process data and tracked termly submission of data from schools and Raising Voices program team, as well as collation and storage of paper copies. Raising Voices intervention delivery data were entered on to standardised excel entry sheets by programme staff, copies of the school Good School term action plans were collected from schools by Program Officers and number of activities listed on the action plans captured on to an excel tracking form by Study Process Monitor and all other process data were collected on paper and double entered on to Epidata. The Study Manager oversaw data entry and the study London based Data Manager ran all data comparison and double entry cleaning reports. The study endline survey data, including Toolkit exposure, were captured on programmed tablet computers with algorithms designed to eliminate erroneous skips. All further data management and analysis was performed in Stata/IC 13.1.

### Analysis

The sample size calculation for the main trial is describe in the main trial paper [4]. All process evaluation analysis is exploratory and therefore no sample size calculation relating to process outcomes are specified for these analyses. All analysis was conducted using Stata/IC 13.1. Endline survey data from the twenty-one intervention schools for both students (*n* = 1921) and staff (*n* = 286) were used for analysis. Missing data from baseline and endline surveys were very low (less than 1% for all measures), however there were more missing data for routine implementation measures collected by schools. Data quality issues, including missing data for process measures, are fully described in Table 2.1 in Additional file 2.

#### Implementation process and endline violence in schools’

To describe the overall implementation of the Toolkit in schools, we calculated school level mean or median values of each measure, along with standard deviations or inter-quartile ranges. To describe the variation in levels of violence in the intervention schools, we calculated the mean percentage of students who experienced any physical violence from staff in the last week, at baseline and endline, along with 95% Confidence intervals.

#### Factors associated with students’ toolkit exposure

To explore whether student’s endline Toolkit exposure was associated with attending a school with “low”, “medium”, or “high” Toolkit implementation, two unadjusted linear regression models are fitted, accounting for school clustering by fitting school as a random effect (Table 1). The two models explore if students had a higher mean Toolkit exposure score (outcome) if they attended a school that reported more (a) planned and (b) completed Toolkit activities, over the implementation period.

**Table 1:** Are students attending schools that implement a higher number of school-led Toolkit activities more exposed to the Toolkit?

	Unadjusted mean increase in student individual Toolkit exposure (95% CI)
Model a) Students in schools reporting low, medium or high number of *planned* school-led Toolkit activities, over four terms of implementation:	
Number of students in regression model	*n* = 1921
Students in schools reporting:	
Low number Toolkit activities planned	ref
Medium number Toolkit activities planned	0.14 (0.05-0.22) 0.003
High number Toolkit activities planned	0.27 (0.15-0.38) < 0.001
Model b) Students in schools reporting low, medium or high number of *completed* school-led activities, over four implementation terms:	
Number of students in regression model	*n* = 1921
Students in schools reporting:	
Low number of completed Toolkit activities	ref
Medium number of completed Toolkit activities	0.30 (0.20-0.39) < 0.001
High number of completed Toolkit activities	0.23 (0.11-0.34) < 0.001

Notation: Linear regression models showing crude associations between students categorised as attending schools reporting low, medium or high levels of Toolkit implementation measured by number of a) planned Toolkit activities and b) completed Toolkit activities, and association with students self-reported exposure to the Toolkit intervention. School level clustering was accounted for by modelling school as a random effect

Legend: Students in schools that reported low, medium or high *planned* school-led Toolkit activities prospectively on their termly action plans (total number planned for the four school terms over the 18 month implementation period), where: low = less than or equal to 30 planned activities, medium = 31 to 45 planned activities and high = 46 or more planned activities. Students in schools that reported a low, medium or high number of *completed* school-led Toolkit activities by the end of the four terms over 18 month implementation period, where: low = less than or equal to 14 completed activities, medium = 15 to 22 completed activities, high = 23 or more completed activities

To explore which student characteristics were associated with student’s Toolkit exposure a linear regression model was fitted, adjusting for school clustering. Choice of factors explored was informed by the conceptual framework for this analysis shown in Additional file 3. Factors that were found to be significantly associated with Toolkit exposure (*p*-value < 0.05) were retained and included in a multivariable model. The reporting of any functional difficulty was identified a priori as a potential important influencing factor and was included in the multivariable analysis despite no significant crude association. Pre-hypothesised interactions between explanatory variables (sex and number of meals eaten, and sex and mental health) were investigated by including interaction terms in the models. Due to evidence of a non-normal distribution of the student’s Toolkit exposure measure, non-parametric bootstrapping with 2000 repetitions was used to estimate bias corrected confidence intervals (Table 2).

**Table 2:** Student factors associated with level of student Toolkit exposure

	Unadjusted mean difference in student Toolkit exposure (95% CI) *P*-value	Adjusted mean difference in student Toolkit exposure (95% CI) *P*-value
Number of students in model	*n* = 1921	*n* = 1914 (a)
Student in current school for full Toolkit implementation period	0.25 (0.14-0.35) < 0.001	0.24 (0.14-0.34) < 0.001
School grade 2014:		
Primary 5	ref	ref
Primary 6	0.05 (−0.03 to 0.14) 0.211	0.04 (− 0.05 to 0.12) 0.431
Primary 7	0.30 (0.20 to 0.39) < 0.001	0.23 (0.14 to 0.32) < 0.001
Poorer mental health	−0.42 (− 0.56 to − 0.27) < 0.001	−0.36 (− 0.51 to − 0.20) < 0.001
Female students	−0.18 (− 0.26 to − 0.10) < 0.001	−0.18 (− 0.25 to − 0.10) < 0.001
Three or more meals eaten yesterday	0.10 (0.03 to 0.18) 0.009	0.07 (− 0.01 to 0.15) 0.084
Absent from school one or more days in last week	−0.12 (− 0.20 to − 0.02) 0.022	−0.09 (− 0.18 to − 0.01) 0.064
Any self-reported functional difficulty	0.03 (− 0.05 to 0.12) 0.455	0.09 (0.00 to 0.18) 0.046
Any other violence experienced in the last year(b)	− 0.10 (− 0.18 to − 0.02) 0.014	−0.05 (− 0.13 to 0.03) 0.206

Notation: *CI* Confidence Interval. (a) Missing data: six students’ responses to being absent from school in the last week and one student response to number of meals eaten yesterday. (b) Any other violence experienced in the last year besides physical violence from school staff

Legend: Linear regression models show individual student factors crude associations with Toolkit exposure and a fully adjusted model that includes all other factors as co-variates, both models account for school clustering by fitting school as a fixed effect

#### Factors associated with physical violence at endline

Spearman’s Rank correlation coefficient was used to examine if schools’ level of Toolkit implementation was correlated with the prevalence of violence in schools. Schools were ranked separately by total number of support visits by Raising Voices program staff, number of school-led Toolkit activities planned and reported as completed, number of Toolkit elements observed in place in school and by school mean of aggregate student, staff and teacher Toolkit exposure. Correlation of each measure with prevalence of physical violence in schools at endline; and change in violence between baseline and endline was explored (Table 3).

**Table 3:** Are school-level process measures correlated with school prevalence of physical violence against students’ from staff at endline and change in prevalence over the Toolkit implementation period

		School endline physical violence prevalence (a)	Change in school physical violence prevalence (b)
School-level process measures	School-level mean (sd), full range	Spearman’s Rank Correlation Coefficient (rho) (*p*-value)	Spearman’s Rank correlation Coefficient (rho) (*p*-value)
Number of schools	21	21	21
Delivery of intervention			
Total number of Raising Voices support visits to school.	8.76 (1.5), 6-12	0.06 (0.797)	0.18 (0.443)
School-led implementation			
Total number of planned school-led activities.	36.95 (9.09), 18-52	0.06 (0.791)	0.09 (0.694)
Total number completed school-led activities reported.	19.33 (6.44), 6-36	0.23 (0.308)	0.12 (0.615)
School adoption			
Toolkit structural elements observed in place, last term of school implementation (count: 0-14).	8.19 (2.25), 4-12	0.30 (0.181)	−0.50 (0.021)
Toolkit reach			
School mean of student Toolkit exposure (count: 0-10)	8.51 (0.45) 7.55-9.39	−0.31 (0.172)	0.35 (0.125)
School mean of staff Toolkit exposure (count: 0-11).	10.03 (0.70) 8.25-11	0.18 (0.442)	−0.05 (0.816)
School mean of teacher Toolkit exposure (count: 0-11).	10.42 (0.48) 9.29-11	−0.23 (0.313)	0.48 (0.029)

A full description of each school-level process measure is provided in Additional file 2

(a) school prevalence of violence at endline: percentage of students reporting physical violence from staff in the last week post intervention – a negative correlation indicates schools with a higher process measure level correlates with schools that have a smaller proportion of students experiencing violence in school

(b) change in school prevalence of violence over implementation period: calculated as endline minus baseline percentage of students reporting physical violence from staff in the last week – a positive correlation indicates schools with a higher implementation process measure correlates to a larger decrease in violence between baseline and end-line

To explore whether student’s Toolkit exposure to different components of the intervention, measured by the continuous factor scores for each factor grouping `active groups’, `classroom rules’, `tools’ and `materials’, are associated with student reports of physical violence from staff in the last week logistic regression models were fitted, accounting for school clustering (Table 4). Similarly, logistic regression models were fitted, adjusted for school clustering, to explore associations between staff Toolkit exposure and reported use of violence against students in the last week and last term (Table 5). Lastly, to explore which student characteristics were associated with students’ self-reported physical violence in the last week from staff, logistic regression models were fitted adjusting for school. Choice of factors explored was based on associations found to be important at baseline and drawing on the conceptual framework for this analysis (Additional file 3). All factors explored for bivariate association are listed in Additional file 4. Factors that were found to be significantly associated with self-reported physical violence (*p* < 0.05) were retained and included in a multivariable model that included a-priori students’ exposure to the Toolkit. Pre-hypothesised interactions between sex, mental health, any other violence in the past year and number of meals eaten yesterday, were investigated by including interaction terms in models.

**Table 4:** Student Toolkit exposure measures associations with physical violence from staff experienced by students in the last week

Student Toolkit exposure measures	Physical violence from staff in last week (students self-reports)
	Unadjusted Odds Ratio (95% CI) *p*-value
Total students in models	*n* = 1921
Exposure to Toolkit (total factor score)	0.76 (0.67-0.86) < 0.001
Toolkit exposure factor groupings:	
Active Groups	0.60 (0.47-0.78) < 0.001
Classroom Rules	0.59 (0.42-0.82) < 0.001
Tools	0.80 (0.56-1.14) 0.223
Materials	0.62 (0.45-0.85) 0.003

Notation: *CI* confidence interval. Legend: Logistic regression model of association between student Toolkit exposure and physical violence from staff, self-reported by students. School clustering has been accounted for by modelling school as a fixed effect. Question items in each factor grouping are presented in Fig. 2b

**Table 5:** Toolkit exposure and self-reported use of physical violence against students in the last week and last term, presented for all school staff and restricted to teaching staff only

Staff and teachers Toolkit exposure measures	Physical violence use against students In the last week (staff self-reports)	Physical violence use against students In the last term (staff self-reports)
	Unadjusted Odds Ratio (95% CI) *p*-value	Unadjusted Odds Ratio (95% CI) *p*-value
Total staff in model	*n* = 283	*n* = 283
Staff Toolkit exposure (count 0-11)	1.0 (0.84-1.21) 0.912	0.95 (0.85-1.07) 0.428
Total teachers in model	*n* = 246	*n* = 246
Teachers Toolkit exposure (count 0-11)	0.84 (0.66-1.07) 0.148	0.77 (0.61-0.98) 0.031

Legend: mixed-effect logistic regression models of association between all school staff and teacher only Toolkit exposure and self-reported physical violence used against students. School clustering has been accounted for by modelling school as a random effect

## Results

### Toolkit implementation: Delivery, school-led implementation, adoption and reach

All twenty-one intervention schools completed the six steps of the Toolkit intervention (Additional file 1), although Raising Voices program officers reported that the intensity and quality of each step varied between schools. School implementation measures are summarised in Fig. 2. On average Raising Voices delivery of support visits took place twice per school term and this was similar between schools. The number of planned and reported school-led activities implemented throughout the whole 18 months (four school terms) implementation period varied between schools, ranging from 18 to 52 and 6 to 36 respectively. Little variation in schools reach to students was seen with schools’ average student Toolkit exposure at endline ranging between 8 and 9 out of 10 exposure questions. School averages of staff Toolkit exposure varied between 8 and 11 out of 11 questions.

### Variation in the prevalence of physical violence in intervention schools at endline

**Fig. 3:**
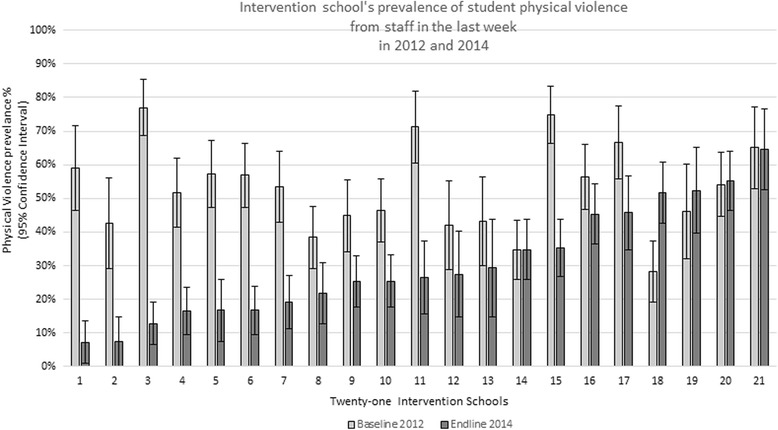
Prevalence of physical violence in intervention schools at baseline and endline Legend: Intervention school-level of physical violence from staff in the last week, reported by students, graph presenting baseline and end-line school means and 95% confidence intervals.

At the end of Toolkit implementation, intervention schools had varying proportions of students reporting physical violence from staff in the last week, ranging from 7.25% (95%CI: 0.97%-13.53%) to 64.62% (95%CI: 52.67%-76.56%) (Fig. 3). Although the Toolkit intervention has been proven to bring about a large average reduction of physical violence in schools, five of the intervention schools remained at similar levels or increased in prevalence of violence at endline compared to baseline, although only one school had a statistically significant increase in violence. In contrast, twelve of the remaining seventeen schools showed a statistically significant decrease in prevalence over the implementation period.

### Factors associated with students’ Toolkit exposure

As expected, student exposure to the Toolkit at endline was associated with attending a school that had reported a higher number of school-led Toolkit activities implemented over the intervention period. Students in schools with a `high’ or `medium’ number of activities planned had a higher mean exposure score compared to those students in schools with a `low’ number planned. A similar association is seen for students attending schools that report more of the Toolkit activities completed, with students in these schools having a higher mean exposure score compared to those in `low’ implementing schools (Table 1).

When investigating individual student’s characteristics and Toolkit exposure, students that had been in the current school for the full implementation period and those students in a higher school grade, had increased odds of exposure to the Toolkit. Girls were much less likely to be exposed than boys. Children experiencing more mental health difficulties were the group of students least likely to be exposed (Table 2).

### School and individual factors associated with physical violence from staff in schools’ at endline

Schools where teachers reported more exposure to the Toolkit had larger decreases in prevalence of school violence between baseline and endline (Table 3). One unexpected observation was that schools with an increased number of Toolkit elements present during the last term of implementation, had a smaller decrease in violence over the implementation period. No other school process measures were significantly correlated with school level violence at endline or decrease in prevalence of violence over the implementation period.

Table 4 shows that students with increased exposure to the Toolkit have a 24% reduction in odds of experiencing physical violence irrespective of which intervention school they attend. In terms of exposure to specific Toolkit processes, participation in Good School `active groups’, `classroom rules’, and Toolkit `materials’ were each independently associated with reduced odds of experiencing violence, whereas awareness of Toolkit `tools’ did not show a significant association.

Table 5 shows that teachers who were more exposed to the Toolkit reported less use of physical violence against students over the last week and last term, although this was only statistically significant over the past school term period.

Table 6 shows that after accounting for individual level exposure to Toolkit activities, girls, students reporting difficulties with self-care (such as washing or dressing), mental health difficulties and those who experienced other violence within the last 12 months, remained at increased risk of experiencing physical violence from staff after the intervention. Having eaten three or more meals the previous day was associated with lower odds of violence.

**Table 6:** Student factors associated with experience of physical violence from staff at endline

Student factors	Unadjusted Physical violence from staff towards students.	Adjusted Physical violence from staff towards students.
	OR (95% CI) *p*-value	aOR (95% CI) *p*-value
Number of students in model	*n* = 1921	*n* = 1920
Exposure to Toolkit (factor score)	0.76 (0.67-0.86) < 0.001	0.82 (0.72-0.93) 0.001
Female sex	1.38 (1.12-1.70) 0.002	1.31 (1.05-1.62) 0.015
Self-reported functional difficulty with memory and/or concentration	1.38 (1.04-1.84) 0.028	NA (1)
Self-reported functional difficulty with self-care (e.g. washing)	3.68 (1.51-8.95) 0.004	3.36 (1.31-8.60) 0.011
Eaten three or more meals yesterday	0.67 (0.53-0.84) < 0.001	0.74 (0.59-0.94) 0.012
Any other violence experienced in the last year (2)	2.27 (1.82-2.84) < 0.001	2.00 (1.60-2.52) < 0.001
Poorer mental health (3)	3.69 (2.45-5.57) < 0.001	2.51 (1.63-3.85) < 0.001

Notation: *aOR* adjusted odds ratio, *CI* confidence interval, *NA* Not Applicable. (1) Self-reported functional difficulty with memory and/or concentration removed from the final multivariate model due to co-linearity with the mental health measure. (2) Any other violence experience in the last year besides physical violence from school staff. (3) Poorer mental health ranging from 0 (low difficulties) to 2 (high difficulties)

Legend: unadjusted and adjusted logistic regression models are presented, both accounting for school clustering by fitting school as a fixed effect. Adjusted model includes all other student factors as covariates in the model

## Discussion

### Summary of main findings

The intervention delivery, in terms of technical support visits to schools from Raising Voices program staff, was similar across schools. In contrast, we observed large variation between schools in Toolkit implementation and adoption measured by number of school-led Toolkit activities planned and reported as completed by schools and in the number of Toolkit structural elements observed in place in each school. Despite variation in levels of school-led implementation, at the end of the implementation period we found high levels of Toolkit reach as measured by school mean aggregates of students and staff exposure to the Toolkit, with relatively little variation across schools. Regardless which school they attended, girls, lower grade students and students with mental health difficulties were less exposed to the intervention. In terms of intervention outcomes, although there was a large average reduction in physical violence, the reduction and prevalence at endline varied widely across schools, ranging from 7% to 65% prevalence. Even after the intervention and adjusting for individual Toolkit exposure, girls, students with mental health difficulties, who experienced other violence, had eaten fewer meals or had difficulties with self-care, were at higher risk of violence compared with other students.

### Strengths and limitations

Like all studies, this process evaluation has strengths and limitations. We used a combination of data collected through a rigorous program of research, and routine monitoring data collected by schools themselves as they would do in the absence of formal research. Data on Toolkit activities planned and completed activities reported by schools were incomplete, and in some cases whole term data were not available from schools. Therefore, these measures may not reflect accurately the number of Toolkit activities in all of the schools. This highlights the need for simple tools and process data embedded in the programme implementation. Measures developed for this study had not been fully tested to determine validity and reliability, however all questions were piloted prior to use. Measures development, construction and data quality issues including estimated internal reliability are documented in Additional file 2. Staff and students responded to interviewer-administered questionnaires during baseline and endline surveys. Like all self-reported measures, there may be some social desirability bias in responses. For teachers, we would expect those more exposed to the Toolkit to report using less violence. However, we would expect a bias in the opposite direction for students, where students who are more exposed to the intervention report more violence experience, which could dilute the effect. Strengths of this evaluation include high student and staff response rate and triangulation of wide range of data sources, use of an independent study process monitor and prospective data collection of process measures.

### Why did the intervention work better in some schools than others?

Taken together, our results suggest that increased exposure to the intervention was the main driver of larger intervention effects. This is true for both increased teacher exposure, and increased student exposure, which supports the Toolkit’s holistic model of engagement with multiple actors within a school to try to engender school-wide change. These results also highlight the importance of on-going training and activities to ensure newly transferred teachers and students are exposed to, and invested in, the Good School Toolkit intervention.

Counter-intuitively, schools with more Toolkit structural elements observed in place by our Study Process Monitor in the final term of the intervention implementation had smaller decreases in violence. This might be explained by a ‘last push’ in schools that were slower to implement. This may have resulted in more visible elements in the final term, without full engagement in the program of work required to sustain these elements or investment in the underlying change. None of the other implementation measures captured at the school level were associated with changes in school violence. We are, however, limited by our relatively small sample size of 21 schools.

In terms of exposure to specific Toolkit processes, participation in ‘active groups’, ‘classroom rules’, and Toolkit ‘materials’ were each independently associated with reduced violence, whereas awareness of ‘Tools’ alone was not. However, qualitatively we found that tools such as the ‘wall of fame’ were perceived positively by students and staff suggesting these are important in promoting reward and praise in schools [12]. In summary, all Toolkit processes seem to be important to bring about change, a finding that supports the idea that multiple and repeated engagement with Toolkit ideas contributes to intervention effectiveness.

Two schools that represent unexpected outcomes are shown on Fig. 3: school number 21 had the highest prevalence of violence at endline and shows no change over time, and school number 18 had a significant increase in violence at endline compared to baseline. Through our program monitoring, we are aware that both of these schools had changes in staff during the implementation of the Good School Toolkit. Anecdotally, it is possible that new staff may be less invested in the program, or in some cases even reverse policy on corporal punishment or dismantle the school wide intervention—shifts Raising Voices has experienced in other schools following staff turnover. This highlights the importance of strong leadership and ownership for the programme to remain successful and stresses the need for early identification of schools requiring additional on-going support. Raising Voices also reported that motivation of the Good School teacher protagonists was also an important factor influencing schools sustained implementation. This suggests that identifying and building on protagonists’ motivation may also be important for an effective program.

The Good School Toolkit intervention includes activities that foster a supportive school environment, aim to challenge negative social norms, and encourage student participation and confidence. Nevertheless, we saw that girls and students with mental health difficulties were less exposed to the Toolkit, irrespective of which school they attend. Conversely, and in line with the inclusive nature of the intervention, students reporting functional difficulties (for example, with sight or hearing), who were more absent from school, who had experienced other violence and eaten fewer meals, reported the same levels of exposure to the Toolkit as other students in their schools. These findings could indicate that there is something within all schools, reflecting broader societal and gender norms, that is preventing girls and students experiencing mental health difficulties from participating in school Toolkit activities. Also, the difference observed in Toolkit exposure for girls, compared to boys, could help explain the main study finding that the intervention was slightly more effective in reducing violence in boys overall [4].

### Which students remain at higher risk of violence after the intervention?

Thirty-one percent of students in intervention schools still experienced physical violence from staff in the last week at endline, demonstrating that even after a highly effective intervention some children were still more at risk of violence compared to their peers. Even after accounting for level of exposure to the intervention, girls, students reporting difficulty with self-care, students who had eaten fewer meals, reported more mental health difficulties and those who experienced other violence besides physical violence from staff in the last year, remained at higher risk of physical violence from staff after the intervention.

The underlying reasons why girls might participate less in Toolkit activities, and remain at higher risk of physical violence from school staff even if they do participate in Toolkit activities, is an area requiring further investigation. Our findings may reflect the need for a social norm shift and sustained school-wide cultural change to address negative gender norms. In addition, the Toolkit may benefit from additional activities and content intentionally designed to enhance participation of these groups. This is in line with findings from other school-based intervention studies in Sub-Saharan Africa. A review of HIV prevention programs in youth in Sub-Saharan Africa that included twenty studies delivered in schools, or schools and community, concluded that “attention should go to studying implementation difficulties, sex differences in responses to interventions and determinants of exposure to interventions” [26]. The need for behaviour change at cultural level was highlighted in a sexual reproductive health school-based intervention in Tanzania, where authors emphasised the need to train and monitor teachers to “have supportive relationships with pupils, boost pupil confidence, encourage critical thinking, challenge dominant gender norms, and not engage in physical or sexual abuse.” [27]. While this quantitative evaluation supports the idea that the Good School Toolkit can bring about school cultural change around the use of violence, we also show that this is not universal to all schools and that harmful norms around violence use against some students remain irrespective of which school the student attends. Although gender equity is implicit in many of the processes and the design of the Toolkit, there may be value in emphasising and making it more explicit to teacher and students, in doing so highlighting Toolkit activities that specifically support gender equality and address negative gender norms.

Student risk factors for violence might reflect the circumstances that influence their likelihood of being physically punished at school. For example, students who have eaten fewer meals might be hungry and this could trigger punishment for having less attention in class. There is some evidence of behavioural and attention problems among hungry children from studies in the United States [28–30]. Students with mental health difficulties might also have difficulty concentrating and might display behaviours that could be seen as disruptive or challenging to teachers who may not have the tools or techniques to deal with children exhibiting these difficulties [31, 32]. This is particularly true in a context with limited teaching resources and large classroom sizes [12, 33] and suggests that positive discipline alternatives to physical punishment may not be well applied or may not be sufficient to prevent violence in some circumstances. Such circumstances may challenge teachers who are still transitioning to non-violent approaches to maintain discipline in their school. Teacher capacity-building around the extra support and skills required for some students in the classroom learning environment is a potential area to focus Toolkit intervention activities.

Our results draw attention to children that may have complex issues, including being poly-victimised, having difficult home environments and dealing with a variety of mental health difficulties[1, 34–36], highlighting the need for strengthening the intervention around building sustained capacity within the school system that recognises children experiencing overlapping vulnerabilities. The layered supportive environment that the Toolkit fosters might be one of the very few opportunities for marginalised children to be involved in a positive school programme, where they can be supported to form better relationships and improve communication - skills that can help build confidence and resilience that influence their choices and future life trajectories.

### Can we develop indicators for programmers to monitor effectiveness?

Collecting data on program implementation, as an indicator of intervention effectiveness, can be useful for future scale-up efforts [8, 37]. The most useful indicators would be school-level, and easily captured during routine implementation monitoring. Unfortunately, none of our implementation measures collected at the school level were associated with the change in school violence over the intervention period or the prevalence measured at endline directly after intervention. These results should be interpreted with caution, as we have low power to quantitatively detect effects across only 21 intervention schools. The lack of association may also be due to the limitations of the monitoring data we collected. Data collected from schools as part of the implementation of the Toolkit had low levels of completeness, and this may have masked a real association between these measures and intervention effectiveness. We also might not have tracked important process indicators. For example, school enforcement of policies or standards promoted by administration are difficult to measure, but may be important predictors of intervention impact. Hence, refinement and reliability testing of in-school assessments, that are in line with the intervention theory of change, could be a useful addition for future program monitoring. This also underscores the importance of capturing qualitative information to understand school context as well as expert programmer’s knowledge of school specific issues relating to effective implementation of the Toolkit.

Our individual-level measures of exposure to the Toolkit were associated with intervention effect, as would be expected. School-level aggregate of teacher’s exposure was also associated with larger reductions in school violence over the implementation period. This may be a potential indicator that could be used by program implementers—although it would require surveying teachers. In addition, our results indicate that monitoring the number of school-led Toolkit activities planned each term could be a simple way to use routine programme data to identify low implementing schools. However, our results highlight that none of the process measures investigated are good indicators of overall intervention effectiveness, and should therefore not be interpreted in the same way as data on violence outcomes.

## Conclusion

Even though the intervention is highly effective at reducing violence against children in school, we found that some schools require additional support to bring about effective and sustained change. It may be possible to increase the effectiveness of the Toolkit by increasing student and teacher exposure. The next layer of Good School programme refinement should attempt to engage with children who were less exposed—in particular, girls, those with poorer mental health and in lower school grade.

## Additional files

Additional file 1: Good School Toolkit description (DOCX 22 kb)

Additional file 2: Good Schools Study process evaluation outcome and process measures (DOCX 44 kb)

Additional file 3: Summary of all student factors explored in analysis (DOCX 17 kb)

Additional file 4: Conceptual frameworks for process evaluation analysis (DOCX 170 kb)
