# Supplementary material for: Implementation of the Good School Toolkit in Uganda: a quantitative process evaluation of a successful violence prevention program
Source: BMC Public Health. 2018 May 9;18:608. doi: 10.1186/s12889-018-5462-1 (PMC5941678; doi:10.1186/s12889-018-5462-1)
Supplement: Supplementary file 1 — Good School Toolkit description (DOCX 22 kb) [file 12889_2018_5462_MOESM1_ESM.docx]

**Annex 1: Good School Toolkit description**

Figure 1.0. The Good School Toolkit intervention by Raising Voices

| **Standard headings to describe intervention** | **Good School Toolkit intervention description** |
| --- | --- |
| **WHY** Describe any rationale, theory, or goal of the elements essential to the intervention. | The goal of the intervention is to foster change of operational culture at the school level. The Toolkit draws on the Transtheoretical Model [[28](http://www.trialsjournal.com/content/14/1/232#B28)], and contains behavioural change techniques that have been shown to be effective in a variety of fields [[25](http://www.trialsjournal.com/content/14/1/232#B25)] and have been included in interventions to change teacher behaviour in primary schools[[22](http://www.trialsjournal.com/content/14/1/232#B22),[27](http://www.trialsjournal.com/content/14/1/232#B27)] and reduce perpetration of intimate partner violence [[26](http://www.trialsjournal.com/content/14/1/232#B26)] |
| **WHAT** |  |
| Materials: Describe any physical or informational materials used in the intervention, including those provided to participants or used in intervention delivery or in training of intervention providers. Provide information on where the materials can be accessed (e.g. online appendix, URL). | The intervention support materials consist of books, booklets, posters and facilitation guides for 60 different activities. These activities are related to creating a better learning environment, respecting each other, understanding power relationships, using non-violent discipline, and to improving teaching techniques. All materials are publicly available at  [www.raisingvoices.org](http://www.raisingvoices.org). |
| Procedures: Describe each of the procedures, activities, and/or processes used in the intervention, including any enabling or support activities. | The Toolkit has six steps which engage teachers, students, administration, and parents to reflect on how they can promote quality of education in their school.  Specific behaviour change techniques for staff, students and administration include: setting school-wide goals, developing action plans with specific dates for deliverables, encouraging empathy by facilitating reflection on experiences of violence, providing new knowledge on alternative non-violent discipline, and to provide opportunities to practice new behavioural skills. Schools are encouraged to self-monitor their progress according to their action plans. Reinforcement of new information and ideas feedback on progress and modeling of new techniques and behaviours is provided by visits from the Raising Voices team, and also within school by ‘protagonists’ to their peers as they gain new knowledge and skills. Schools reward successful achievement of their goals and action plan deliverables by creating celebrations. Social support for behavioural change is also created because the intervention engages multiple groups within a school (teachers, administration, students, and also parents) to change ideas and attitudes. |
| **WHO PROVIDED** |  |
| For each category of intervention provider (e.g. psychologist, nursing assistant), describe their expertise, background and any specific training given. | Raising voices staff members provide direct one on one support to two key students and two key staff ‘protagonists’ in each school. Raising voices staff members are trained facilitators and advocates, and have received approximately 100 hours of training with individualized coaching support to understand the ideas and content of the Toolkit. The key protagonists’ in each school are not required to have any specific background or training. |
| **HOW** |  |
| Describe the modes of delivery (e.g. face-to-face or by some other mechanism, such as internet or telephone) of the intervention and whether it was provided individually or in a group. | The protagonists attend a three day residential workshop run by Raising Voices. Raising Voices staff members provide direct one on one support in the form of in-person visits and telephone calls to staff protagonists, and in-person visits to student protagonists. Staff and student protagonists conduct face-to-face activities with other staff and students in their school, mainly in groups. |
| **WHERE** |  |
| Describe the type(s) of location(s) where the intervention occurred, including any necessary infrastructure or relevant features. | Activities were conducted in schools. Some activities involved creating a better school environment by painting murals on in school walls, and hanging codes of conduct in visible places. However the intervention does not require any physical infrastructure. |
| **WHEN and HOW MUCH** |  |
| Describe the number of times the intervention was delivered and over what period of time including the number of sessions, their schedule, and their duration, intensity or dose. | Each school received an inception visit made by RV for two hours where all staff learned about the Toolkit. Once the school was committed to implementing the Toolkit, two protagonists were identified who attended three day residential workshop. During that workshop they developed an action plan which then RV staff monitored on a monthly basis. Raising voices staff made in-person visits to protagonists in each school on a quarterly basis and telephoned staff members approximately monthly, although this varied to slightly depending on need. The Toolkit itself is designed to be implemented and a flexible fashion, and there are no prescribed number of activities or set schedule upon which they should be implemented. |
| **TAILORING and modifications** |  |
| If the intervention was planned to be personalised, titrated or adapted, then describe what, why, when, and how. | The Toolkit consists of a number of different activities, and it is intended so that schools pick and choose which activities they implement. However they must implement a certain minimum number of activities prior to formally progress to the next step. |

Figure 1.1. A summary of the Good School Toolkit implementation steps

***Good School Toolkit Steps***

***Step One: Your Team & Network***

- Schools identify key protagonists at school and create their Good School Committee to build school-wide support for the process (*pre-contemplation*)

***Step Two: Preparing for Change***

- Baseline measurements gather information on each schools’ starting point, and school leaders cultivate interest among parents, the community and local education officials (*contemplation*)

***Step Three: Good Teachers & Teaching***

- A school-wide reflection on teacher-student relationships provides a renewed sense of teacher roles, increased professional support, and new approaches for positive student engagement (*preparing for action*)

***Step Four: Positive Discipline***

- Schools reflect on how violence manifests and establish a new school culture by exploring positive disciplinary methods to create students who believe in themselves (*action*)

***Step Five: Good Learning Environment***

- Schools reflect on what a good learning environment looks like and work with all stakeholders to foster a psychological sense of safety and inclusion (*maintenance of action*)

***Step Six: Good Administration & the Future***

- The work of the preceding steps is celebrated and consolidated through reflection and transfer of leadership to the school administration (*consolidation of gains*)
