# Supplementary material for: Implementation of the Good School Toolkit in Uganda: a quantitative process evaluation of a successful violence prevention program
Source: BMC Public Health. 2018 May 9;18:608. doi: 10.1186/s12889-018-5462-1 (PMC5941678; doi:10.1186/s12889-018-5462-1)
Supplement: Supplementary file 2 — Good Schools Study process evaluation outcome and process measures (DOCX 44 kb) [file 12889_2018_5462_MOESM2_ESM.docx]

**Annex 2: Good Schools Study process evaluation outcome and process measures**

**Table 2.0. Process evaluation outcomes measures**

| **Outcome**  **variable name** | **Instrument, Items and comments on variable construction** | **Coding** | **Number/denominator, %** | |
| --- | --- | --- | --- | --- |
| **Physical violence** | | | | |
| Physical violence from staff against students, self-reported by students in intervention schools  Time frame: past week | International Society for the Prevention of Child Abuse and Neglect Screening Tool-Child Institutional [18] (ICAST-CI), wording adapted and modified to include time frames and piloted prior to use in student surveys.  Has a school staff member: hurt you or caused pain to you? Slapped you with a hand on your face or head as punishment? Slapped you with a hand on your arm or hand? Twisted your ear as punishment? Twisted your arm as punishment? Pulled your hair as punishment? Hit you by throwing an object at you? Hit you with a closed fist? Hit you with a stick? Caned you? Kicked you? Knocked you on the head as punishment? Made you dig, slash a field, or do other labour as punishment? Hit your fingers or hands with an object as punishment? Crushed your fingers or hands as punishment? Made you stand /kneel in a way that hurts to punish you? Made you stay outside for example in the heat or rain to punish you? Burnt you as punishment? Taken your food away from you as punishment? Forced you to do something that was dangerous? Choked you? Tied you up with a rope or belt at school? Tried to cut you purposefully with a sharp object? Severely beat you up? | Coded 1 if answered yes to any of the items; 0 if answered no to all items. | 595/1921,  30.97% | |
| Physical violence (use of), self-reported by staff members  Time frame: past week and past term | Adapted from the International Society for the Prevention of Child Abuse and Neglect Screening Tool-Child Institutional (ICAST-CI) [18], modified to include time frames and piloted prior to use in staff surveys.  What are the methods of physical discipline you have used with students? Have you ever: Slapped them with a hand on their face or head as punishment? Twisted their ear as punishment? Twisted their arm as punishment? Pulled their hair as punishment? Hit them by throwing an object at them? Hit them with a closed fist? Hit them with a stick? Caned them? Kicked them? Knocked them on the head as punishment? Made them dig, slash a field, or do other labour as punishment? Hit their fingers or hands with an object as punishment? Crushed their fingers or hands as punishment? Made them stand/kneel in a way that hurts to punish them? Made them stay outside for example in the heat or rain to punish them? Burnt them as punishment? Taken their food away as punishment? Forced them to do something that was dangerous? Choked them? Tied them up (with a rope or belt) at school? Tried to cut them purposefully with a sharp object? Made them roll over on the ground until they were dizzy as punishment? | Coded 1 if answered yes to any of the items; 0 if answered no to all items. | Past week all staff:  44/283,  15·55%  Past term all staff:  91/283,  32.16% | |
| Physical violence (use of), self-reported by teaching staff only  Time frame: past week and past term | Instrument and Items as above for physical violence (use of), self-reported by staff members restricted to teaching staff only.  Teaching staff includes head teachers, senior men/women teachers and general teachers, other non-teaching staff roles include cooks and administration roles. Teaching staff make up 87% (246 of the total 283 staff). | Coding as above. | Past week teachers only:  44/246,  17.89%  Past term teachers only:  86/246,  34.96% | |
| **Student Toolkit exposure** | | | | |
| Student exposure to Toolkit,  factor score | Toolkit exposure questions were developed by study team and Raising Voices specifically to measure exposure to components of the Toolkit. Question wording was pre-tested among a small number of students and added to the end-line student survey. Each of the ten questions shown below had a “yes” or “no” response. Cronbach's alpha of 0.56 and therefore exploratory factor analysis was performed on student exposure responses. Exploratory factor analysis with tetrachoric correction was performed using the ten exposure questions and questions fell in to four factor groupings. A continuous variable was generated by summing the four factor scores generated from exploratory factor analysis to give an overall factor score “student Toolkit exposure score”. Factor grouping and questions:  **Factor group1: Active groups:**   1. My school has a pupils court that is different than the prefects council or discipline committee, 2. My schools has a Good Schools pupils committee, 3. I have participated in a meeting/Any activity organised by the Good Schools pupils committee.   **Factor group 2: Classroom Rules:**   1. My school has written classroom rules and regulations for how pupils should behave, 2. My class participated in making up these written rules, 3. These written rules are displayed in my classroom where pupils can see them.   **Factor group 3: Tools:**   1. My school has a wall of fame for pupils, 2. My school has a suggestion box where pupils can put ideas.   **Factor group 4: Materials:**   1. In my school, I have seen a poster or booklet about Good Schools, like this (child is shown example materials from the Toolkit pack), 2. In my school, I have participated in a discussion about Good Schools posters or booklets. | Total factor score  Generated | Median: 2.74,  IQR: 2.23-3.61  Range: -0.91-4.52 |  |

**Table 2.1. Process measures and exposure questions**

| **Process measure** | **Instrument, Items** | **Coding** | **Data Quality Issues** |
| --- | --- | --- | --- |
| **Delivery of intervention to schools by Raising Voices (RV)** | |  |  |
| Total RV school visits. | Total number of RV Program Officers support visits to school. Total visits counted over the whole implementation period. Data captured by RV Program Officers prospectively on to standardised electronic excel data collection sheet. | Total count over 4 implementation school terms | None, presented back to the RV team for face validation. |
| **School-led implementation of Toolkit activities** | | | |
| Total planned school-led activities. | Total number of planned school-led Toolkit activities. Schools complete and submit a termly Toolkit action plan and the total number of activities listed on the action plans over the four school terms of Toolkit implementation are counted. Data captured by schools on to standardised paper termly action plans as part of routine Good School program in-school planning and monitoring process. | Total count over 4 implementation school terms | Missing data. Three schools did not provide the action plan on one term and two schools for two terms-counted as 0 activities planned for missing term data. In Term 3 all schools used a standard pre-set (pre printed) 12 activity action plan. |
| Total reported school-led in school Toolkit activities. | Total number school-led Toolkit activities reported. Schools submitted standardised monitoring reports for each Toolkit activity recorded during the four school term implementation and the total number received counted. | Total count over 4 implementation school terms | Missing data. One schools did not report any activities for one term and two school did not report any for two terms- counted as 0 activities for those terms. |
| Further data quality note on planned and reported Toolkit in school activities | The percentage (%) of planned activities reported (total number of activities reported/total planned activities) for all schools was 44%, 57%, 39% and 86% for implementation terms 1-4, respectively. Individual schools reported between 18%-92% of planned activities completed over the full period, this must be interpreted with caution due to the missing data. The known zero returns and potential for other missing data on termly planned and/or reported activities means these measures may not represent actual planning and activities that took place in some schools. The Study Process Monitor undertook multiple attempts to retrieve plans and reports from schools throughout and at the end of the study. Hence, these measures are based on real life data, with the understanding that they may not represent accurately number of activities that actually took place in schools. Missing data results in a lower total count, meaning that both measures also reflect poor monitoring and reporting of the Toolkit implementation by schools. During the fourth implementation term only (School year 2014 term 1) the Study Process Monitor made weekly monitored phone calls to each school to check on number of planned Toolkit activities that had taken place, as documented on the school Toolkit term action plan, and to encourage completion and reporting of each activity – this may account for a higher proportion of 86% activities planned that were reported via individual paper activities reports completed by schools and submitted to the Study Process Monitor. | | |
| **School adoption of Toolkit elements Toolkit** | | | |
| Adoption of Toolkit structural elements | Adoption of Toolkit structural elements measure is a count of 0 to 15 based on responses to 15 questions asked during school wide assessment in the final term of the four Toolkit implementation terms (school term 1, 2014). Positive responses “yes” are counted as 1. A positive response corresponds to affirming that the material/item was provided by Raising Voices and that it had been observed in the school by the Study Process Monitor at time of school assessment visit.  Questions:   1. Are the grounds tidy?, 2. Written code of conduct for students posted where observable to students? 3. Written code of conduct for staff posted where observable to staff and students? 4. Any other written rules/policies posted where observable to staff (e.g. classroom rules)? 5. Any other written rules/policies posted where observable to students? 6. Any posters about the various aspects of a Good School? 7. Is there a Good School wall mural? 8. Is there a ‘Wall of Fame’ or ‘Board of Fame’? 9. Something to celebrate ‘Student of the Month’? 10. A suggestion box visible to students and staff? 11. An idea board? 12. Is children’s work displayed on classroom walls or anywhere else on school compound? 13. Is there a garden? 14. Are there any signs with relevant school messages, such as ‘no littering’, ‘respect the environment’ etc.?   Not included in score:   1. 7. Any name tags or ribbons worn by students or teachers to promote a Good School? | Total count  (0-14)  Number  of schools, %  21, 100%  0, 0%  3, 14%  4, 19%  9, 43%  21, 100%  20, 95%  8, 38%  4, 19%  20, 95%  13, 62%  9, 43%  13, 62%  18, 86%  0, 0% | Structural element 1 and 6 were observed in all intervention schools in the final implementation term. Structural element 2 and 15 were not observed in any intervention schools. 15 was not part of the final intervention package therefore removed from total count. There may have been some changes in wording of Toolkit tools such as “ideas board” which effected accurate data collection.  Cronbach's alpha 0.67. |
| **Toolkit reach to students and teachers in schools** | | | |
| School mean student Toolkit exposure (reach). | Student Toolkit exposure questions were developed by study team and Raising Voices specifically to measure exposure to components of the Toolkit. Questions were piloted among students and added to the end-line student survey. Student exposure is the count of individual student positive “yes” responses to the following ten Toolkit exposure questions 1-10, showed below. School mean student exposure is calculated as the school mean of student exposure count. These are the same questions used to generate student individual exposure scores through factor analysis, as detailed above.  Student Toolkit exposure questions:   1. My school has a pupils court that is different than the prefects council or discipline committee, 2. My school has a Good Schools pupils committee, 3. I have participated in a meeting/Any activity organised by the Good Schools pupils committee, 4. My school has written classroom rules and regulations for how pupils should behave, 5. My class participated in making up these written rules, 6. These written rules are displayed in my classroom where pupils can see them, 7. My school has a wall of fame for pupils, 8. My school has a suggestion box where pupils can put ideas. 9. In my school, I have seen a poster or booklet about Good Schools, like this (child is shown example materials from the Toolkit pack), 10. In my school, I have participated in a discussion about Good Schools posters or booklets.   Not included in count:   1. I have participated in the student court | School mean of students’ exposure count 0-10, higher the more exposure to the Toolkit. | One question “I have participated in the student court” was not used in total count as we were unable to interpret whether child was disciplined through court, been a witness in the court or sat as a court member.  Cronbach's alpha 0.56 |
| School mean staff and teacher Toolkit exposure. | Staff Toolkit exposure questions were developed by the study team and Raising Voices specifically to measure exposure to components of the Toolkit. Questions were piloted among school staff and added to the end-line staff survey. School mean staff exposure is calculated as the school mean of exposure count for all staff interviewed in that school. School mean teacher exposure is calculated as the school mean for teaching staff only.  Teaching staff comprise 87% of the total staff respondents, other staff include cooks, administration and other non-teaching roles.  Staff and teacher Toolkit exposure: Staff and teacher exposure is the count of individual positive “yes” responses to the following eleven Toolkit exposure questions:   1. My school has a pupils court that is different than the prefects council or discipline committee, 2. My schools has a Good Schools pupils committee, 3. I have participated in a meeting/any activity organised by the Good Schools pupils committee, 4. My school has written classroom rules and regulations for how pupils should behave, 5. My pupils participated in making up these written rules, 6. These written rules are displayed in my classroom where pupils can see them, 7. My school has a wall of fame for pupils, 8. My school has a suggestion box where pupils can put ideas, 9. In my school, I have seen a poster or booklet about Good Schools, like this (show materials), 10. In my school, I have participated in a discussion about Good Schools posters or booklets, 11. My school has a Good Schools staff committee | School mean of staff and teachers only exposure sores. Score 0-11, higher the more exposure to the Toolkit. | Some questions were not designed well to pick up non-teachers exposure to the Toolkit (e.g. cooks and administrators). For example: “My pupils participated in making up these written rules.” as certain staff would not be routinely interacting with students in this way. Hence overall staff exposure is presented in addition to exposure and analysis restricted to teaching staff only.  Cronbach's alpha 0.72. |
