# Supplementary material for: Implementation of the Good School Toolkit in Uganda: a quantitative process evaluation of a successful violence prevention program
Source: BMC Public Health. 2018 May 9;18:608. doi: 10.1186/s12889-018-5462-1 (PMC5941678; doi:10.1186/s12889-018-5462-1)
Supplement: Supplementary file 3 — Summary of all student factors explored in analysis (DOCX 17 kb) [file 12889_2018_5462_MOESM3_ESM.docx]

**Annex 3: Summary of all student factors explored in analysis**

**Table 3.0. Summary of student factors**

All factors listed below were explored for crude association with student Toolkit exposure and physical violence from staff.

| **Variable** | **Measure, coding** | **Number, %** | **Missing data** |
| --- | --- | --- | --- |
| Total | - | 1921 | - |
| Sex: | Categorical:  Male: 0  Female: 1 | 885, 46.07  1036,53.93 | 0 |
| School grade | Categorical:  Primary 5: 0  Primary 6: 1  Primary 7: 2 | 748, 38.94  654, 34.04  519, 27.02 | 0 |
| In current school for full implementation period | Dichotomous:  No: 0  Yes: 1 | 370, 19.26  1551, 80.74 | 0 |
| Three or more meals eaten yesterday | Dichotomous:  Less than three: 0  Three or more: 1 | 1117, 58.15  803, 41.80 | 1 |
| Number of hours worked outside of school | Ordered categorical:  Less than 1 hour: 0  One to two hours: 1  More than two hours: 2 | 665. 34.87  910, 47.41  332, 17.41 | 14 |
| Number of children sharing sleeping area | Dichotomous:  None or one other: 0  Two or more: 1 | 731, 38.05  1190, 61.95 | 0 |
| Transport to school | Categorical  Other: 0  Walking alone: 1  Walking with someone you know: 2  Board at school: 3 | 100, 5.24  433, 22.67  1249, 65.39  128, 6.70 | 11 |
| Absent one or more days in the past week | Dichotomous:  Missed no school days in last week: 0  Missed one day or more in the last week: 1 | 1531, 79.70  384, 20.05 | 6 |
| Any other violence experienced in the last year. | Dichotomous:  Other violence in last  year: 1  No other violence in last year: 0  Any “yes” response to any violent act from non-staff (peers, caregivers and other adult relatives, others) or “yes” to any emotional or sexual violence from school staff, in the last 12 months (ref other violence paper). | 972, 50.60  949, 49.40 | 0 |
| Any self-reported functional difficulty | Dichotomous:  No functional difficulty 0  Any functional difficulty 1  Any functional difficulty is constructed as 1 if one or more of the six functional difficulties listed below are coded 1, and 0 if none are coded 1. | 1464, 76.21  457, 23.79 | 0 |
| Separate functional difficulties 1-6: | Separate functional difficulty modelled as a binary variable, coded 1 if answered: “some difficulty”, “a lot of difficulty”, or “you cannot do this at all” and coded 0 if “none”. |  |  |
| 1. Seeing | Do you have difficulty seeing, even if wearing glasses  No difficulty 0  Any difficulty 1 | 1840, 95.78  81, 4.22 | 0 |
| 1. Hearing | Do you have difficulty hearing, even if using a hearing aid?  No difficulty 0  Any difficulty 1 | 1864, 97.03  57, 2.97 | 0 |
| 1. Concentration and/or memory | Do you have difficulty remembering or concentrating?  No difficulty 0  Any difficulty 1 | 1645, 85.63  276, 14.37 | 0 |
| 1. Self-care | Do you have difficulty with self-care, such as washing all over or dressing?  No difficulty 0  Any difficulty 1 | 1898, 98.80  23, 1.20 | 0 |
| 1. Movement | Do you have difficulty walking or climbing steps?  No difficulty 0  Any difficulty 1 | 1866, 97.14  55, 2.86 | 0 |
| 1. Communicating | Using your usual language, do you have difficulty communicating, for example understanding, or being understood?  No difficulty 0  Any difficulty 1 | 1868, 97.24  53, 2.76 | 0 |

| **Variable** | **Measure, coding** | **Median, IQR** | **Missing data** |
| --- | --- | --- | --- |
| Age | Continuous | 13, 12-14 | 0 |
| Mental health symptoms | Continuous:  Strengths and Difficulties Questionnaire (SDQ)^19^  (20 items). Total difficulties score, divided by the number of completed items. Modelled as a continuous variable. Range 0 (no difficulties) to 2 (high difficulties). | 0.4, 0.25-0.60 | 0 |
