# Supplementary material for: Implementation of the Good School Toolkit in Uganda: a quantitative process evaluation of a successful violence prevention program
Source: BMC Public Health. 2018 May 9;18:608. doi: 10.1186/s12889-018-5462-1 (PMC5941678; doi:10.1186/s12889-018-5462-1)
Supplement: Supplementary file 4 — Conceptual frameworks for process evaluation analysis (DOCX 170 kb) [file 12889_2018_5462_MOESM4_ESM.docx]

Annex 4: Conceptual frameworks for process evaluation analysis

Figure 4.0. Conceptual framework for exploring student factors that may influence Toolkit exposure


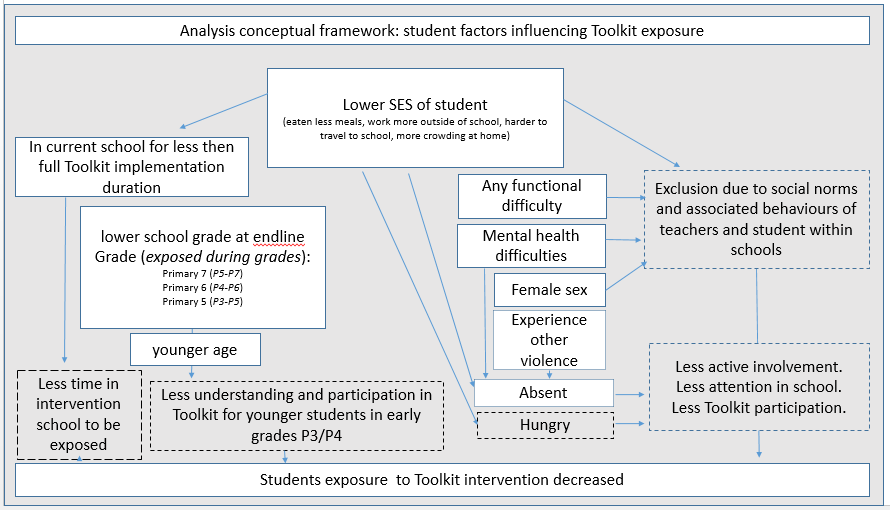


Legend: The information in white boxes relate to student factor variables explored in the analysis. The boxes outlined with dashed lines are hypothesised conceptual consequences that may influence student’s exposure to Toolkit. The blue arrows denote hypothesized direction of simplified relationships between explored factors and concepts influencing exposure to Toolkit.

Figure 4.1. Conceptual framework for student factors influencing Toolkit exposure


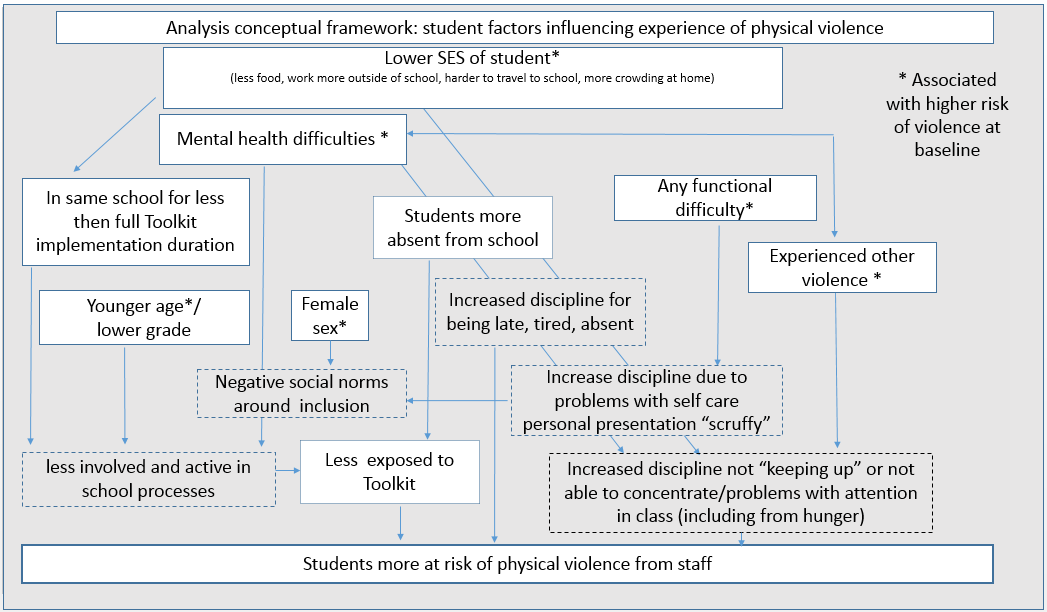


Legend: The information in white boxes relate to student factor variables explored in the analysis. The boxes outlined with dashed lines are hypothesised conceptual consequences that may influence student’s experience of violence from staff. The blue arrows denote hypothesized direction of simplified relationships between explored factors and concepts that might influence student’s experience of physical violence from staff.
